# Lymphangiogenesis in myocardial remodelling after infarction

**DOI:** 10.1111/j.1365-2559.2007.02785.x

**Published:** 2007-09-01

**Authors:** Y Ishikawa, Y Akishima-Fukasawa, K Ito, Y Akasaka, M Tanaka, R Shimokawa, M Kimura-Matsumoto, H Morita, S Sato, I Kamata, T Ishii

**Affiliations:** Department of Pathology, Toho University School of Medicine Tokyo, Japan; 1 Pathology Division, National Cancer Centre Research Institute Tokyo, Japan; 2 Pathology Division, Tokyo Metropolitan Hiroo Hospital Tokyo, Japan; 3 Pathology Division, Omori Red Cross Hospital Tokyo, Japan; 4 Department of Gastroenterology and Hepatology, Toho University Medical Centre Omori Hospital Tokyo, Japan

**Keywords:** blood vessel, lymphangiogenesis, lymphatic vessel, myocardial infarction, myocardial remodelling, vascular endothelial growth factor-C

## Abstract

Ishikawa Y, Akishima-Fukasawa Y, Ito K, Akasaka Y, Tanaka M, Shimokawa R, Kimura-Matsumoto M, Morita H, Sato S, Kamata I & Ishii T (2007) *Histopathology***51,** 345–353

**Lymphangiogenesis in myocardial remodelling after infarction**

**Aims:**

The lymphatic system is involved in fluid homeostasis of the cardiac interstitium, but lymphangiogenesis in myocardial remodelling has not previously been examined histopathologically. The aim was to investigate by D2-40 immunohistochemistry the sequential changes in lymphatic distribution in the process of myocardial remodelling after myocardial infarction (MI).

**Methods and results:**

Myocardial tissues in various phases of healing after MI were obtained from 40 autopsied hearts. D2-40+ lymphatic vessel density (LD) and CD34+ blood vessel density (BD) in the lesion were determined. BD decreased with advance of myocardial necrosis, subsequently increased at the early stage of granulation and thereafter decreased with the progression of scar formation. In contrast, lymphatic vessels were not detected in lesions with coagulation necrosis, and newly formed lymphatics first appeared in the early stages of granulation. A subsequent increase in LD was demonstrated in the late stages of granulation, and lymphatics remained up to the scar phase. Vascular endothelial growth factor-C was consistently expressed in viable cardiomyocytes around the lesion in all of these stages.

**Conclusion:**

In myocardial remodelling after MI, lymphangiogenesis lags behind blood vessel angiogenesis; newly formed lymphatics may be involved mainly in the maturation of fibrosis and scar formation through the drainage of excessive proteins and fluid.

## Introduction

Myocardial fluid homeostasis is modulated by the balance between fluid filtration into the myocardial interstitium from blood capillaries and its removal from the interstitium via the lymphatic system.^1^ Impairment of cardiac lymphatic flow due to several pathological states, such as ventricular fibrillation, increased superior vena caval pressure, pulmonary artery hypertension and myocardial ischaemia, results in excess fluid accumulation within the myocardial interstitium,^1^ which often leads to interstitial fibrosis, pericardial effusion and subsequent myocardial dysfunction.^2^,^3^ In the mammalian heart, cardiac lymph is drained from the subendocardial lymphatic plexus to the subepicardial lymphatic plexus through intramyocardial lymphatic channels.^3^ These cardiac lymphatic networks in human and animals have repeatedly been investigated in detail by observations using dye-injection techniques, electron microscopy and lymphangiography.^3^–^5^ However, these techniques are inappropriate for histopathological observation of the lymphatic system in formalin-fixed paraffin-embedded tissues obtained from myocardial tissues.

In the human heart, myocardial remodelling after myocardial infarction (MI) results in scar formation through several sequential stages of myocardial necrosis, granulation and fibrosis.^6^ In the process of myocardial remodelling, the viable cardiomyocytes around the lesion express cytoprotective proteins and cytokines which facilitate the healing of the affected lesion.^7^,^8^ In addition, prolyl 4-hydroxylase expressed in the cardiomyocytes around the lesion as well as fibronectin and adiponectin expressed in the affected area effectively contribute to interstitial remodelling.^9^ Myocardial remodelling is, thus, dependent on various contributors, and vascular endothelial growth factor (VEGF) is critical for angiogenesis in the healing area.^10^ VEGF is promptly expressed in the surviving cardiomyocytes around the infracted lesion after the onset of MI^7^ and angiogenesis in the lesion begins at 4–5 h and continues up to day 90.^11^ The process of angiogenesis in the infarcted area has been investigated sporadically,^12^,^13^ but lymphangiogenesis in the healing process of MI has not previously been examined. This is probably due to the lack of a reliable marker that can distinguish the lymphatic endothelium from the blood capillary endothelium.

In recent years, lymphatic endothelial cell-specific proteins have been identified, including VEGF receptor-3, lymphatic endothelial hyaluronan receptor-1 (LYVE-1), podoplanin, prox-1, D6 and D2-40.^14^,^15^ We have also examined lymphatic distribution in various human tissues using polyclonal LYVE-1 rabbit antibody and D2-40 antibody.^14^,^16^ In the heart, a recent study has reported lymphatic morphology and density in transplanted cardiac tissues by immunohistochemistry using anti-flt-4 (VEGF receptor-3) antibody,^17^ but did not include lymphangiogenesis in MI lesions. In this study, we investigated fluctuation of lymphatic distribution in the entire process of myocardial remodelling after the onset of MI by immunohistochemistry using D2-40 antibody, which was also compared with blood vessel distribution as well as VEGF-C expression in the cardiomyocytes around the foci.

## Materials and methods

### Materials

Myocardial tissue was obtained from 40 autopsied patients with acute MI (AMI) and/or old MI (OMI). As normal controls, an additional five cases with no cardiac lesion were used. All cases were autopsied within 4 h postmortem. The MI cases included nine cases of AMI, 15 of OMI and 16 with both AMI and OMI. All MI lesions were recognized in the left ventricular walls. The mean age of the 40 patients with MI was 70.2 ± 9.5 years (range 44–92 years) and 28 were male and 12 female. The five control cases with no cardiac lesion were aged 58–79 years (mean 66.5 ± 8.5 years) consisting of three men and two women.

### Tissue preparation and immunohistochemistry

The 10 normal myocardial tissues were dissected from the left ventricle of five control cases. The lesions in each case of MI were also dissected in the same manner and there were 88 myocardial tissue blocks with MI. All myocardial tissues were fixed in 10% neutral buffered formalin and embedded in paraffin. Thin sections were treated with haematoxylin and eosin (H&E) and Azan–Mallory staining.

Additional subserial sections from all the above paraffin blocks were used for immunohistochemistry. The antibodies used were D2-40 (Signet Laboratory Inc., Dedham, MA, USA), anti-CD34 (DakoCytomation, Carpinteria, CA, USA), anti-α-smooth muscle actin (SMA) (DakoCytomation), anti-CD68 (DakoCytomation) and anti-VEGF-C (Zymed Laboratories Inc., South San Francisco, CA, USA). Immunohistochemistry was performed using the Envision+ kit (DakoCytomation) according to the manufacturer's instructions, except for VEGF-C immunohistochemistry, which was performed with the CSA II kit (DakoCytomation). Sections were visualized by treating the slides with diaminobenzidine-tetrahydrochloride. To verify antibody specificity, sections from each paraffin block were used as negative controls by omitting the primary antibody and replacing it with normal mouse or rabbit immunoglobulin.

### Staging of the lesions

After observation of sections treated with H&E and Azan–Mallory as well SMA and CD68 immunohistochemistry, each MI lesion was classified into one of the seven stages of sequential histopathological change during the healing of MI, as proposed by Lodge-Patch:^6^ Stage I, the earliest changes where stretching and waviness of myocardial fibres are observed and these myocytes have eosinophilic cytoplasm and pyknosis; Stage II, coagulation necrosis of cardiomyocytes with haemorrhage or neutrophil infiltration, but without CD68+ macrophages; Stage III, coagulation necrosis of cardiomyocytes with infiltration of CD68+ macrophages in addition to neutrophils; Stage IV, the early stage of granulation in which fragmented myocytes with coagulation necrosis, many CD68+ macrophages, a few neutrophils and fibroblasts are found; Stage V, mature granulation tissue with CD68+ macrophages and fibroblasts, but without necrotic myocytes or neutrophils; Stage VI, the stage of fibrosis with abundant myofibroblasts positive for SMA, in which the interstitium is weakly stained blue by Azan–Mallory throughout the lesion; and Stage VII, the lesion is replaced with scar tissue with a decrease of myofibroblasts and uniformly stained blue by Azan–Mallory.

Of the 88 myocardial lesions, the number of cases in each stage was nine in Stage I, 18 in Stage II, 13 in Stage III, 11 in Stage IV, six in Stage V, 14 in Stage VI and 17 in Stage VII.

### Statistical analyses

In the normal cases, the sections immunostained with D2-40 antibody were observed by light microscopy at ×200 magnification. Only vessels with a lumen or consisting of more than a single endothelial cell positive for D2-40 were considered as lymphatic vessels. The number of D2-40+ lymphatics in the myocardial interstitium was counted at random in 20 fields and the total number of lymphatics in 20 fields was defined as lymphatic vessel density (LD) in each section. The sections immunopositive for CD34 antibody were observed in the same way as in the count of LD cases. The total number of CD34+ vessels in 20 fields was counted and the number [(the number of CD34+ vessels) − (the number of D2-40+ vessels)] was defined as blood vessel density (BD) in each section, because CD34+ vessels may include both lymphatic and blood vessels.

In MI cases, sections immunopositive for D2-40 antibody were observed in the same way as normal cases and the total number of D2-40+ lymphatics in 20 fields of MI lesions was regarded as the LD in each lesion. The BD in each lesion was also determined by the same methods as in normal cases.

The average value of LD in each categorized stage was statistically compared with those of normal cases and the other stages by Student's *t*-test using the StatView program (SAS Inc., Cary, NC, USA). The average value of BD in each categorized stage was also treated statistically. Differences at *P* < 0.05 were considered to be statistically significant.

## Results

### Distribution of lymphatic and blood vessels and VEGF-C expression in the left ventricle of normal heart

**Figure 1:**
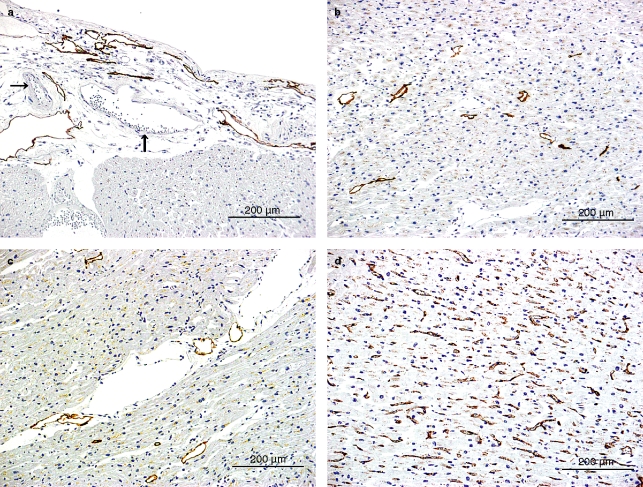
D2-40 (a – c) and CD34 d) immunohistochemistry in the normal heart. **a,** D2-40+ lymphatics are abundant in the subepicardium of the normal heart. Blood vessels indicated by arrows are immunonegative for D2-40. b, Lymphatic capillaries are scattered among cardiomyocytes. **c**, The lumens of lymphatics adjacent to blood vessels are dilated. **d**, CD34+ blood capillaries are numerous among cardiomyocytes.

In normal hearts, D2-40+ lymphatics were frequently observed in the interstitium of the subepicardium (Figure 1a). The interstitium around arteries and veins distributing in the subepicardial adipose tissue also had abundant lymphatics. In the normal myocardium, small lymphatics (lymphatic capillaries) were sporadically scattered among cardiomyocytes (Figure 1b). In the interstitium around arteries and veins running in the myocardium, relatively abundant lymphatics were distributed, and they had larger lumens than lymphatic capillaries existing among myocytes (Figure 1c). In the subendocardium, there were abundant lymphatics. These distribution patterns of lymphatics in the ventricular wall were commonly recognized in the anterior, lateral and posterior walls.

On the other hand, blood vessels were more numerous in the normal myocardium compared with the lymphatics. In the subepicardial region, coronary arteries and veins were found, and a few capillaries positive for CD34 were seen in the interstitium of subepicardial adipose tissue. In the ventricular myocardium, blood capillaries were numerous among cardiomyocytes (Figure 1d).

### Distribution of lymphatic and blood vessels and VEGF-C expression in the myocardium affected by mi

#### Stage I

**Figure 2:**
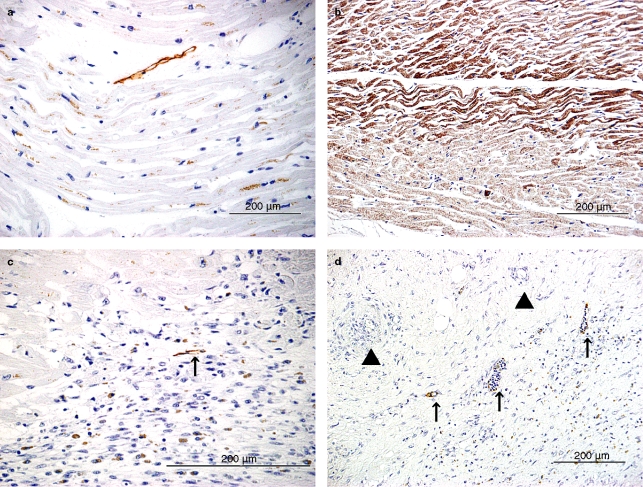
Immunohistochemistry in Stages I (a,b), IV (c) and V (d) a, A D2-40+ lymphatic vessel is recognized in the interstitium of the lesion with wavy myocardial fibres (Stage I). b, The upper half of the figure shows wavy myocardial fibres which are strongly positive for vascular endothelial growth factor-C (VEGF-C). Cardiomyocytes around the lesion (the lower half of the figure) are also weakly immunopositive for VEGF-C. c, In Stage IV, showing the early change of granulation, a small D2-40+ lymphatic vessel (arrow) is shown in the vicinity of the viable cardiomyocytes (the left upper area of the figure). d, In Stage V, showing the mature granulation phase with infiltration of macrophages and lymphocytes, D2-40+ lymphatics (arrows) are scattered. A lymphatic vessel in the centre of the figure contains lymphocytes in the lumen. Arrowheads indicate muscular blood vessels.

The interstitium in this lesion was oedematous and included a few D2-40+ lymphatic capillaries (Figure 2a), but the number of lymphatics seemed to be decreased compared with that in the normal myocardium. VEGF-C was weakly expressed in the viable cardiomyocytes around the lesion as well as being strongly expressed in the affected cardiomyocytes (Figure 2b).

Capillaries positive for CD34 in this lesion were abundant, but there was a mild decrease in their density compared with that in the normal myocardium. Arterioles and venules were intact, as were arteries and veins.

#### Stage II

D2-40+ lymphatic vessels were not seen in the interstitium of the lesion. In addition, the number of capillaries positive for CD34 also decreased compared with Stage I. Endothelial cells of arterioles and venules were barely positive for CD34, and those of arteries and veins were relatively intact. VEGF-C expression was widely demonstrated in the cardiomyocytes around the foci, but not in coagulative myocytes.

#### Stage III

In the interstitium, lymphatic vessels were not seen, nor were blood capillaries; arterioles and venules were rarely detected. Endothelial cells of arteries and veins were barely positive for CD34. VEGF-C was expressed in viable cardiomyocytes around the lesions.

#### Stage IV

A few D2-40+ lymphatics were found in the peripheral region of the affected area (Figure 2c). On the other hand, capillaries positive for CD34 were abundant throughout the affected lesion and a small number of muscular blood vessels showing immunopositivity for SMA were also observed in the peripheral region. VEGF-C was expressed in the cardiomyocytes around the lesion.

#### Stage V

This stage exhibited mature granulation tissue in which CD68+ macrophages and fibroblasts were relatively abundant with a few lymphocytes. The interstitium of this lesion was barely stained light blue with Azan–Mallory. Lymphatics were mainly distributed in the peripheral region of granulation tissue (Figure 2d) and exhibited a dilated structure. In the central area of granulation tissue, lymphatics were scarce and capillary in nature. On the other hand, CD34+ vessels were almost uniformly distributed throughout the lesion and included many muscular blood vessels immunopositive for SMA in addition to blood capillaries. VEGF-C expression was observed in the cardiomyocytes around the lesion.

#### Stage VI

**Figure 3:**
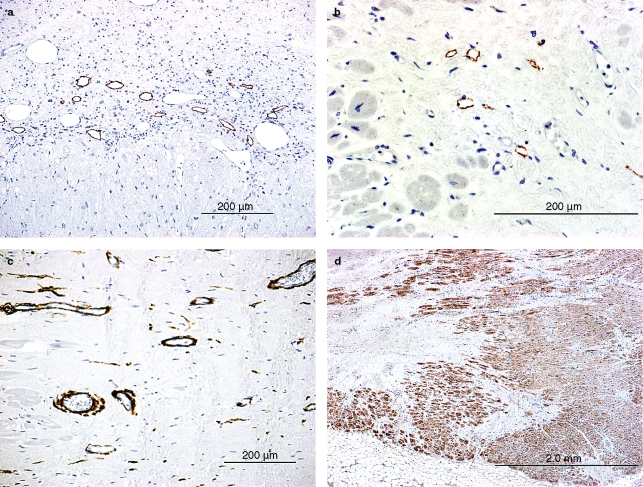
Immunohistochemistry in Stages VI (a) and VII (b**–**d)**a**, The upper half of the figure shows the lesion with fibrosis (Stage VI), in which D2-40+ lymphatics are scattered adjacent to the viable cardiomyocytes (the lower half of the figure). Their lumens are dilated. **b**, In Stage VII, showing scar formation, D2-40+ lymphatics are scattered in the periphery of the lesion. c, In the scar, muscular blood vessels immunopositive for smooth muscle actin are scattered. **d**, Cardiomyocytes around the scar are positive for vascular endothelial growth factor-C.

In these lesions, dilated lymphatics were observed in the peripheral region (Figure 3a). Lymphatic capillaries were also scattered in the lesion and tended to be distributed in the peripheral region.

The number of blood vessels in fibrous lesions decreased compared with Stage V. Most blood vessels observed in this lesion had muscular walls immunopositive for SMA, and blood capillaries were rare. Cardiomyocytes around the lesion were immunopositive for VEGF-C.

#### Stage VII

In a scar lesion, cell components were scarce and the whole area was uniformly stained blue with Azan–Mallory. Lymphatics were sporadically recognized in scar tissue (Figure 3b). In the central region of scar tissue, lymphatics were scarce, as were CD34+ capillaries. The number of muscular blood vessels in scar tissue decreased compared with Stage VI (Figure 3c). However, VEGF-C was expressed in the cardiomyocytes around the lesion, as in the other stages (Figure 3d).

### FLUCTUATION OF LD and BD IN THE HEALING PROCESS OF MI LESIONS

**Figure 4:**
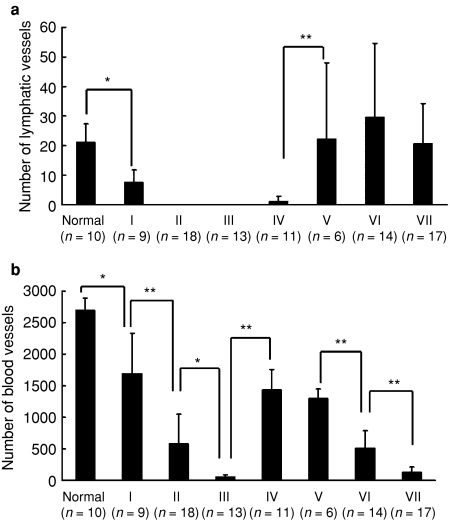
Lymphatic density (LD; a) and blood vessel density (BD; b) in the myocardium. **a**, LDs in the normal myocardium and the seven categorized stages. LD means the total number of D2-40+ lymphatics in 20 fields of view observed by light microscopy at ×200 magnification. The values of LD are 21.2 ± 6.2 in the normal myocardial tissue (Normal), 7.6 ± 4.2 in Stage I, 0 in Stage II, 0 in Stage III, 1.3 ± 1.5 in Stage IV, 22.3 ± 25.7 in Stage V, 29.7 ± 25.0 in Stage VI and 20.8 ± 13.4 in Stage VII. There is no statistical difference between LD values in Stages V, VI and VII. Statistical analyses were performed by Student's *t*-test. *P < 0.0001; **P < 0.05. **b**, Blood vessel density (BD) in the normal myocardium and the seven categorized stages. BD means the total number of CD34+ blood vessels counted by the same methods as the value of lymphatic density. The values of BD are 2693.4 ± 197.0 in Normal, 1689.9 ± 640.7 in Stage I, 571.4 ± 478.3 in Stage II, 51.8 ± 30.5 in Stage III, 1430.8 ± 323.1 in Stage IV, 1292.7 ± 153.1 in Stage V, 509.9 ± 276.3 in Stage VI and 130.5 ± 83.8 in Stage VII. After the onset of myocardial infarction, the BD value decreased up to Stage III. The BD values increased in Stage IV compared with Stage III, but decreased with the advance of the healing process of infarcted lesion up to Stage VII. Statistical analyses were performed by Student's *t*-test. *P < 0.001; **P < 0.0001.

LD in the normal heart and the seven categorized lesions is indicated in Figure 4a. LD in Stage I was significantly smaller than that in the normal myocardium. In Stages II and III, lymphatic vessels were never seen in the lesions, but a few lymphatics were observed in Stage IV. However, LD in Stage IV was significantly less than that in the normal myocardium and Stage I. In Stages V, VI and VII, LD increased significantly compared with Stage IV, but there was no statistical difference in LD between Stages V, VI and VII.

BD in the normal heart and the seven categorized lesions is indicated in Figure 4b. BD in the lesions of all stages was significantly less than that in the normal myocardium. BD decreased with the advance from Stage I up to Stage III. However, BD abruptly increased in Stage IV and subsequently decreased with the healing process of the lesion up to Stage VII.

## Discussion

The present study has shown that lymphatic vessels were not detected in stages with coagulation necrosis, but a few lymphatics first appeared in the peripheral region adjacent to viable myocytes in the early granulation period. LD subsequently increased in the mature granulation period and was thereafter maintained during scar formation. On the other hand, BD decreased in the affected area in stages with coagulation necrosis, but abruptly increased at the early stage of granulation with subsequent decrease with the process of MI healing. Lymphangiogenesis after the onset of MI lagged behind blood vessel angiogenesis, whereas VEGF-C was expressed in the cardiomyocytes around the lesion in all stages of myocardial remodelling. From these results, it can be seen that, during the entire process of MI healing, blood vessels supply the blood and nutrients mainly during the granulation period, but lymphatics participate mainly in fibrosis maturation and scar formation through the drainage of excessive proteins and fluid.

In the normal heart tissue used in the present study, D2-40+ lymphatics were abundant in the subepicardial and subendocardial regions, as well as in the interstitium around the coronary arteries and veins. These findings are consistent with the distribution patterns of cardiac lymphatics confirmed by other methods.^3^ In the light of lymphatic distribution, the abundant lymphatics in the subepicardial region detected by D2-40 immunohistochemistry seem to correspond to the subepicardial lymphatic plexus, and those in the subendocardial region to the subendocardial plexus.^3^ In addition, D2-40 immunohistochemistry in this study detected lymphatic capillaries distributing in the interstitium among the cardiomyocytes, which were far fewer in number compared with BD. Our previous study describing lymphatic distribution in sections of normal renal tissue, and other reports,^18^,^19^ have also demonstrated the usefulness of D2-40 immunohistochemistry for detecting lymphatic capillaries in the renal interstitium.^16^

Myocardial necrosis after the onset of MI begins with a wavy change of myocardial fibres after a few hours, which becomes maximal at about 5–6 days, and the damaged myocardial fibres disappear within about 2 weeks, at which stage granulation occurs.^11^ In this process of myocardial necrosis, extracellular matrices are degraded by inflammatory cell proteases.^20^ From such early events after the onset of MI, it is supposed that pre-existing vascular tissues in the affected area also disintegrate through the influence of ischaemia and inflammatory cells. The present results have clearly demonstrated that BD in the infarcted lesion significantly decreases with the advance of myocardial necrosis from Stage I up to Stage III. In Stage III, which showed complete coagulation necrosis of myocytes with infiltration of neutrophils and macrophages, blood capillaries were not seen, whereas a few muscular blood vessels immunopositive for SMA remained sporadic in the lesions. These muscular vessels should hardly have survived because of the meagre blood supply, as immunopositivity for CD34 was weak. On the other hand, D2-40+ lymphatic vessels were not detected in the affected areas in Stages II and III. The difference of LD and BD in Stages II and III seems to be caused by the structural difference between lymphatic and blood vessels. Lymphatics in the cardiac interstitium consist of single-layered, thin endothelial cells which possess undeveloped basement membrane with no pericytes around them.^3^ These characteristics of lymphatic structures may cause vulnerability to ischaemic damage and/or degradation by proteases, as with blood capillaries.

In the present study, BD surprisingly increased in the early granulation phase (Stage IV), when newly formed blood capillaries were uniformly distributed. This result is similar to those in experimental canine MI models.^21^,^22^ The increase in BD in the granulation phase probably results from the effect of VEGF, because viable cardiomyocytes around the MI lesion promptly express VEGF after the onset of MI.^7^ On the other hand, the present study has revealed only a few lymphatics in the peripheral region adjacent to viable cardiomyocytes in the early granulation phase, whereas VEGF-C was expressed in the lesion promptly after the onset of MI and showed a significant increase in BD in the mature granulation phase (Stage V). In general, lymphangiogenesis develops through budding, sprouting and/or remodelling from the pre-existing vessels, which frequently occur in pathological conditions, such as wound healing, inflammation, lymphoedema and malignant tumours.^23^,^24^ In the process of cutaneous wound healing in the mouse model, lymphatics appear in subcutaneous tissue along the wound edge at 3–5 days after injury.^25^ It is therefore hypothesized that newly formed lymphatics first appear in the peripheral area of the infarcted lesion through sprouting from the pre-existing lymphatics around the lesion, and the proliferation of lymphatics follows after blood vessel angiogenesis.

The occurrence of lymphangiogenesis later than blood vessel angiogenesis has been demonstrated in excisional wound repair^26^ and experimental tumour*s* in the mouse model,^27^ as well as in the present study. The reason why repair-associated lymphangiogenesis occurs after blood vessel angiogenesis remains unknown. Previous reports have disputed that a pre-existing blood vascular bed is necessary to guide lymphangiogenesis,^28^ proposing that, on the contrary, lymphangiogenesis can occur in the absence of a pre-existing vascular bed.^29^ A recent report has emphasized that the organ-specific environment is a major determinant of lymphatic vessel formation,^26^ and another has also suggested that the delay in tumour lymphangiogenesis compared with angiogenesis is due to a lack of lymphatic vessels surrounding the tumour at an early stage.^27^ In the present study, VEGF-C, the most crucial factor in the lymphangiogenic cascade,^23^ was expressed in viable cardiomyocytes around the lesion just after the onset of MI, but the increase of LD in the lesion surprisingly lagged behind blood vessel angiogenesis. This phenomenon is probably due to the difference in proliferative capacity between lymphatic and blood vessel endothelial cells or the lower density of pre-existing lymphatics in the myocardium compared with that of blood vessels.

In the current study, BD significantly decreased with healing maturation from Stage V up to Stage VII, whereas LD showed no significant decrease at these stages. These results indicate that blood vessels function mainly to supply nutrients in the phase of granulation tissue formation after myocardial necrosis, and lymphatics participate mainly in the maturation of fibrosis and/or scar formation through the drainage of excessive proteins and fluid. In general, fibrous tissue is observed at 3 weeks after the onset of MI, with a white, firm scar being formed by 3 months.^6^,^11^ From these descriptions, including our results, it is considered that the role of lymphatics during myocardial healing may be maintained for longer than that of blood vessels and that immunohistochemistry using a lymphatic endothelial marker is useful in investigating the role of lymphatics in cardiac disease.

## Abbreviations

AMI: acute myocardial infarction
BD: blood vessel density
H&E: haematoxylin and eosin
LD: lymphatic density
LYVE-1: lymphatic endothelial hyaluronan receptor-1
MI: myocardial infarction
OMI: old myocardial infarction
SMA: smooth muscle actin
VEGF: vascular endothelial growth factor
